# Case series on 2 cases of macular damage caused by the novel coronavirus: A case report

**DOI:** 10.1097/MD.0000000000041076

**Published:** 2025-01-17

**Authors:** Yong Guo, Chenjun Guo, Yan Li, Yongkang Zhang, Xiaozhan Liu, Houcheng Liang

**Affiliations:** a Xi’an BRIGHT Eye Hospital Group Co Ltd. Xi’an, Shaanxi, China; b The Second Affiliated Hospital of Air Force Military Medical University, Xi’an, Shaanxi, China.

**Keywords:** fundus photography, hormone therapy, macular disease, macular OCT, novel coronavirus infection

## Abstract

**Rationale:**

Macular damage is a rare yet significant ocular complication of coronavirus disease 2019 (COVID-19) infection. This report highlights the clinical features, diagnosis, treatment, and outcomes in 2 cases of COVID-19-associated macular damage, contributing to the understanding of its pathophysiology and management.

**Patient concerns:**

Both patients presented with a sudden onset of visual impairment and black shadows in their central visual fields shortly after contracting COVID-19.

**Diagnoses:**

The first patient, a 15-year-old male, was diagnosed with acute macular neuroretinopathy, evidenced by optical coherence tomography (OCT) imaging showing high-density shadows in the macular area. The second patient, a 28-year-old female, presented with more extensive macular lesions, confirmed via fundus photography and OCT imaging.

**Interventions:**

Both patients received systemic corticosteroid therapy (oral prednisone acetate, 30 mg daily). The male patient was treated for 5 days, while the female patient underwent a 3-day treatment course.

**Outcomes:**

The male patient showed significant improvement in visual acuity, with OCT imaging revealing the resolution of high-density shadows but persistent disruption of the ellipsoid zone. The female patient demonstrated partial improvement in visual symptoms, though residual macular abnormalities remained evident on imaging.

**Lessons:**

COVID-19-associated macular damage appears to involve inflammatory and vascular mechanisms. Early systemic corticosteroid therapy may offer symptomatic relief and partially restore visual function. However, long-term monitoring is essential to address potential residual or progressive retinal damage.

## 1. Introduction

The coronavirus disease 2019 (COVID-19) outbreak that began in December 2019 has had a profound and lasting impact globally. The full extent of this disease is not yet clear, but it has been shown to cause a range of clinical presentations and multi-organ damage that can lead to permanent sequelae. However, ocular complications of COVID-19 are often overlooked. The 2 cases reported in this article were both seen by ophthalmologists due to visual dysfunction. This article analyzes the characteristics and treatment outcomes of these patients, providing a valuable addition to the case data on COVID-19 ocular disease and a basis for further research and treatment of COVID-19 retinal disease. The patient in the 2 case reports has provided informed consent for the publication of this case report. This consent ensures compliance with the CARE guidelines, which mandate the inclusion of a patient consent statement. The complete lack of any consent, as per the CARE guidelines, precludes the publication of this case.

## 2. Case report

**Figure 1.**
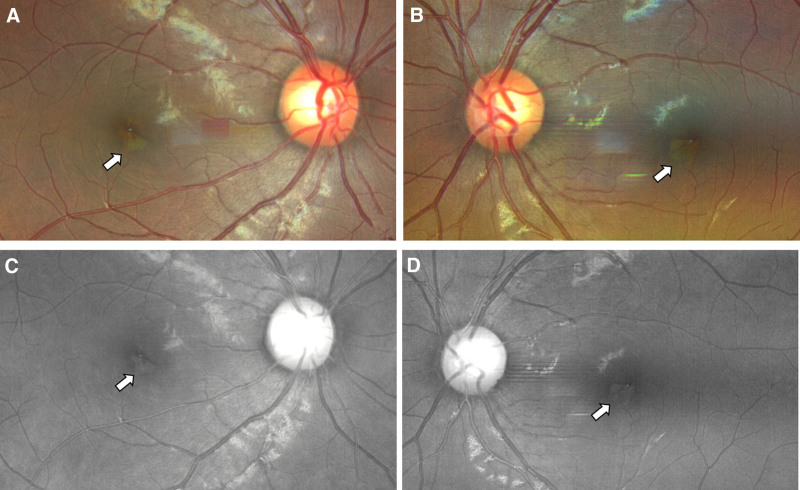
A 15-year-old male with COVID-19 infection shows low-density shadows (arrow) in the fundus after 3 days. Right eye fundus photograph (A), left eye fundus photograph (B), right eye fundus without red-free (C), left eye fundus without red-free (D). COVID-19 = coronavirus disease 2019.

**Figure 2.**
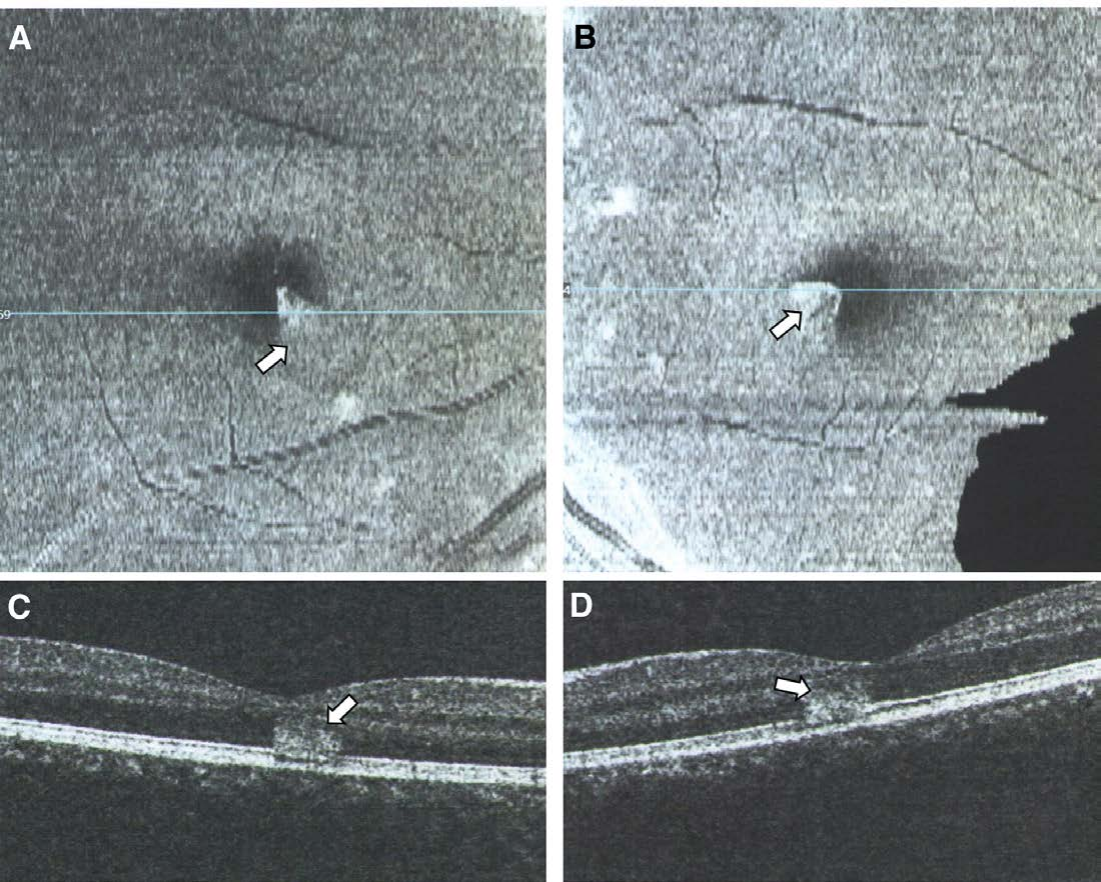
A 15-year-old male with COVID-19 infection shows high-density shadows (arrow) in OCT after 3 days. Right eye enfaces retina surface (A), left eye enface retina surface (B), right eye OCT fovea (C), left eye OCT fovea (D). COVID-19 = coronavirus disease 2019, OCT = optical coherence tomography.

**Figure 3.**
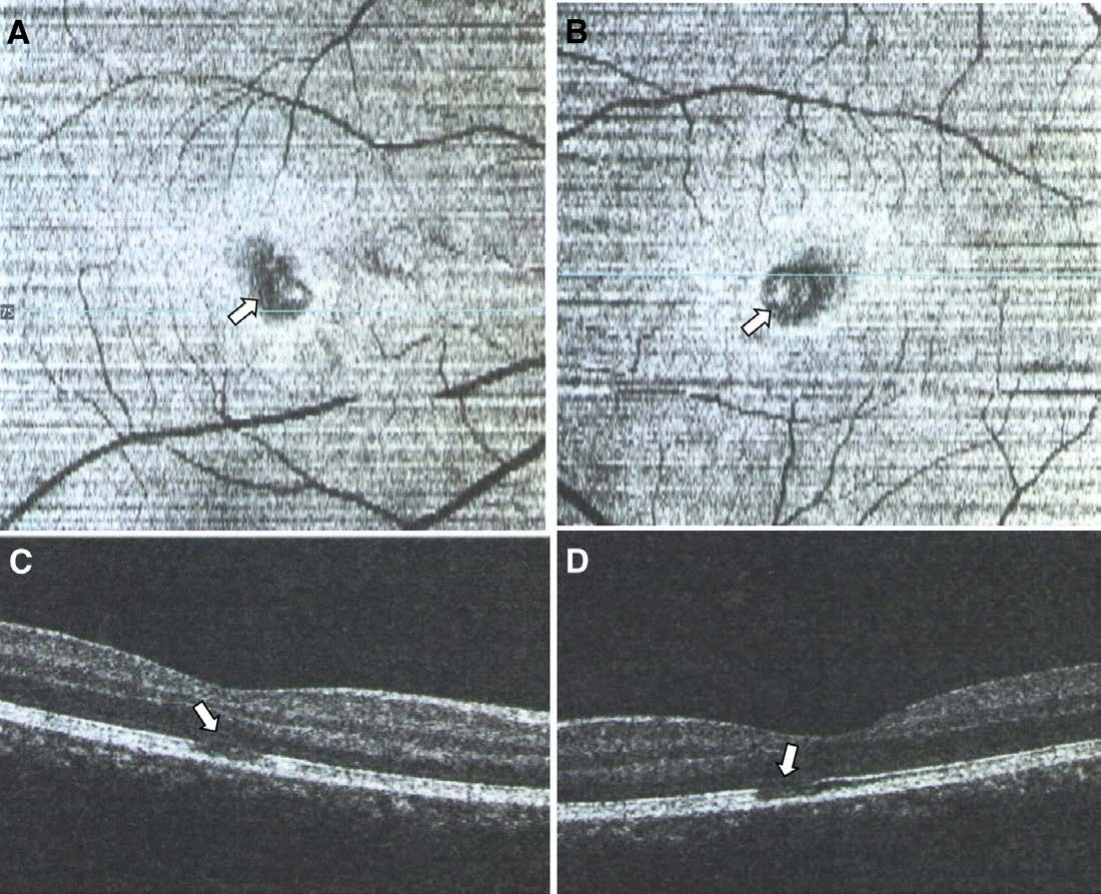
A 15-year-old male with hormone treatment shows a missing ellipsoid zone (arrow) in OCT after 5 days. Right eye enfaces IS/OS-ellipsoid layer (A), left eye enface IS/OS-ellipsoid layer (B), right eye OCT fovea (C), left eye OCT fovea (D). IS/OS = inner segment/outer segment layer, OCT = optical coherence tomography.

Case 1: A 15-year-old male who has been living in Xi’an, Shaanxi, China for a long time, came to our hospital for treatment on December 20, 2022, due to “fever for 2 days and sudden visual impairment with black shadows in front of the eyes for 1 day.” The patient has no history of chronic diseases or drug allergies. He suddenly had a fever 3 days ago, with the highest body temperature of 38.3°C, and the fever subsided 1 day ago, without a sore throat, cough, or other symptoms. Suddenly, the patient’s vision decreased, with a fixed black shadow in the central field of vision. The general examination was negative. Ophthalmologic examination showed no conjunctival congestion, clear cornea, normal anterior chamber depth, no flash or keratic precipitates, round pupils, sensitive light reflex, clear crystalline lens and the vitreous, red retina without hemorrhage or exudation, and central fovea of the macula with less clear reflex, and a triangular small piece of edema with a diameter of 1/6 pupillary distance in the macular area. Whole body examination: COVID-19 nucleic acid test (+) in the throat swab, COVID-19 antigen test (+) in the nasal and throat swab. Special examination: visual acuity Oculus Dexter (OD; right eye) 0.8 Oculus Sinister (OS; left eye) 0.8, no improvement in corrected visual acuity, Heidelberg Retina Angiography examination, both color photograph, and the red-free photograph showed triangular neural epithelial edema below the nasal side in the fovea of both eyes, with the tip of the triangle facing towards the central foveal depression (Fig. 1). Optical coherence tomography (OCT) examination revealed high-density shadows in the form of blocks between the neural epithelial and pigment epithelial layers in the foveal area, without obvious interstitial effusion (Fig. 2). The patient has been given oral prednisolone acetate tablets of 30 mg once a day for 5 days. After 5 days, the patient came for a follow-up visit and reported that his vision had improved significantly compared to before, and the black shadow in front of his eyes still existed but was smaller and lighter in color. Whole body examination: COVID-19 nucleic acid test (+) in the throat swab, COVID-19 antigen test (−) in the nasal and throat swab. Special examination: visual acuity OD 1.0 OS 1.0, no improvement in corrected visual acuity, OCT examination, a high-density shadow in the neural epithelial layer disappeared, and the ellipsoid band in the macular area was interrupted. The enface scan showed a low-density shadow in a small area of the ellipsoid band in the macular area (Fig. 3).

**Figure 4.**
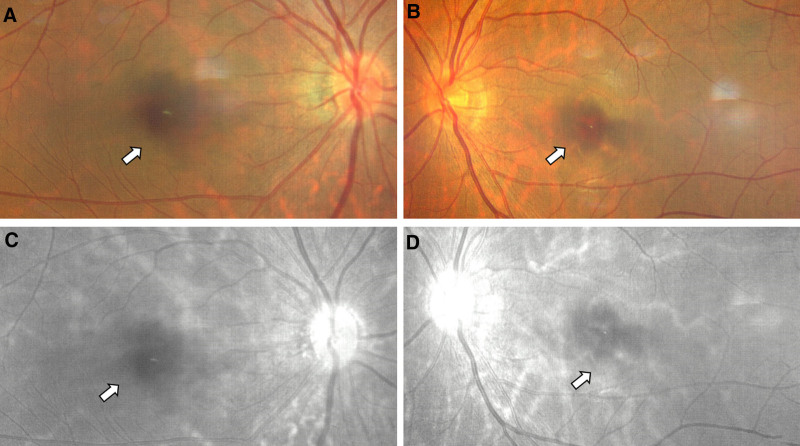
A 28-year-old female with COVID-19 infection shows low-density shadows (arrow) in the fundus after 10 days. Right eye fundus photograph (A), left eye fundus photograph (B), right eye fundus without red-free (C), left eye fundus without red-free (D). COVID-19 = coronavirus disease 2019.

**Figure 5.**
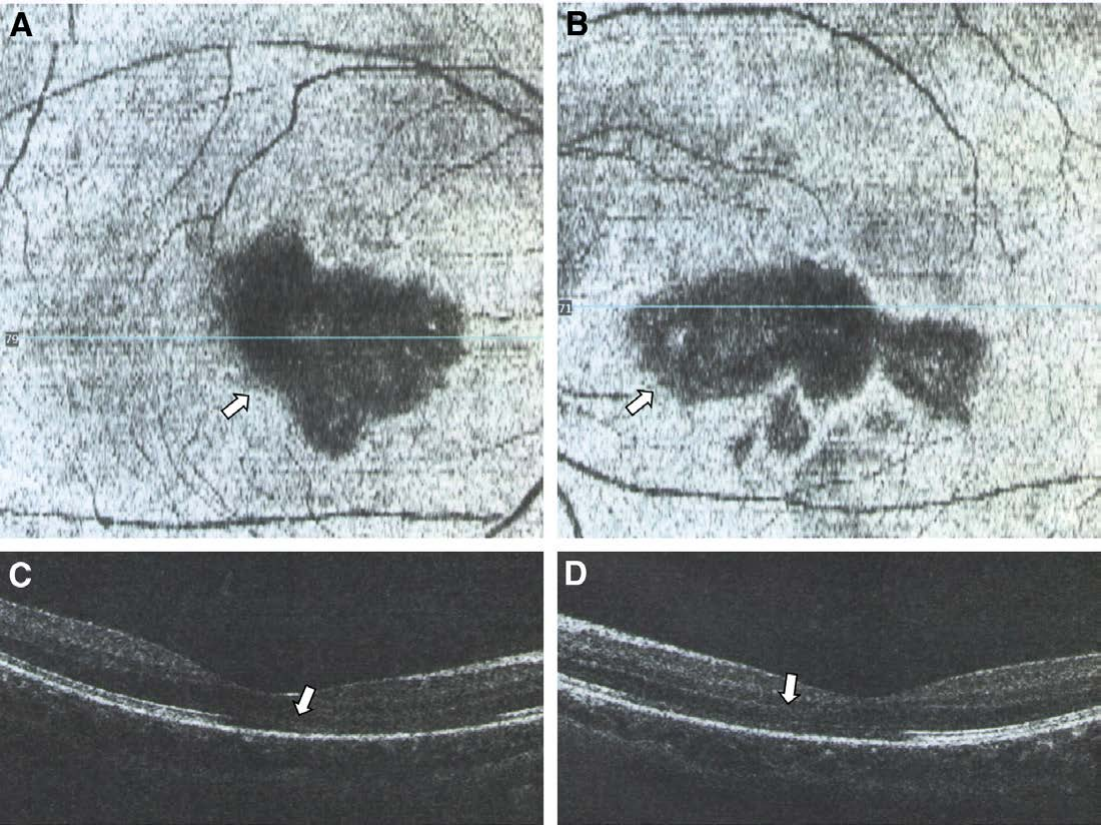
A 28-year-old female with COVID-19 infection shows a missing ellipsoid zone (arrow) in OCT after 10 days. Right eye enfaces IS/OS-ellipsoid layer (A), left eye enface IS/OS-ellipsoid layer (B), right eye OCT fovea (C), left eye OCT fovea (D). COVID-19 = coronavirus disease 2019, IS/OS = inner segment/outer segment layer, OCT = optical coherence tomography

Case 2: Female, 28 years old, a long-term resident of Xi’an, Shaanxi province, China. On January 9, 2023, the patient came to our hospital because of “sudden blurred vision accompanied by black shadows in front of the eyes for 10 days, worsened 1 day ago.” The patient had no history of any chronic diseases or drug allergies. Ten days ago, there was a sudden decrease in vision, and a fixed black shadow appeared in the central visual field. No treatment was given, and the symptoms worsened 1 day ago. The physical examination was negative. The ophthalmic examination revealed no conjunctival congestion, clear cornea, normal anterior chamber, no flashing lights or keratic precipitates, round pupils, sensitive light reflex, clear lens, clear vitreous, red retina without hemorrhage or exudation, poor reflection in the center of the macula, and central macula depression. The general examination revealed positive results for the COVID-19 nucleic acid test from a throat swab and negative results for the COVID-19 antigen test from a nasal swab. The specialized examination revealed visual acuity of OD 0.4 and OS 0.3, with no improvement after correction, wide-angle fundus photography showed large central shadows in both eyes and decreased background reflection (Fig. 4). OCT showed large oval lesions in the macula area in both eyes. The enface scan showed large oval lesions in the macula area (Fig. 5). The patient was given oral prednisone acetate 30 mg once a day, for 3 days, and was advised to come back for a follow-up visit in 3 days.

Written informed consent was obtained from the legal guardians or next of kin of the patients within this specific age group who belong to the vulnerable/minor population.

## 3. Discussion

COVID-19 infection can cause a wide range of systemic multiorgan diseases. Severe Acute Respiratory Syndrome coronavirus 2 (SARS-CoV-2) is the main virus, which enters the body through the angiotensin-converting enzyme 2 on the surface of cells. The locations of angiotensin-converting enzyme 2 in the eye are the conjunctiva, cornea, and retina.^[1,2]^ Cases of conjunctival infection with COVID-19 are the most frequently reported, with an incidence rate of 0.8% to 32%.^[3–6]^ COVID-19-induced viral keratitis has also been occasionally found.^[7,8]^ Although the SARS-CoV-2 virus has been found in the vitreous and retina of COVID-19 deceased patients,^[9,10]^ posterior segment damage, especially retinal disease, caused by COVID-19 infection is still relatively rare. De Figueiredo et al^[11]^ speculated that retinal damage is caused by SARS-CoV-2 infection through the conjunctiva and cornea into the vitreous cavity. However, the 2 patients in this article had no changes in the conjunctiva or cornea, and the retinal damage appeared directly, which does not support this hypothesis.

COVID-19 infection’s most common pathogenesis is causing systemic microvascular injury.^[12]^ Marinho et al,^[12]^ Landecho et al,^[13]^ and Pereira et al^[14]^ have all found small patches of hemorrhages and cotton-wool spots in the fundus of COVID-19-infected patients, and they speculated that it was due to microvascular thrombosis. The 2 patients in this article did not have any hemorrhages, but the macula area had small patches of low-density shadows, and OCT showed elliptical cystoid lesions, consistent with the pathology of microvascular thrombosis. Central retinal artery occlusion (CRAO) and Acute Macular Neuroretinopathy (AMN) are microvascular and deep retinal vessel diseases of the retina. CRAO occurs in the inner nuclear layer, while AMN occurs at the junction of the outer plexiform and outer nuclear layers.^[15]^ The OCT of the 2 patients in this article showed high-density shadows between the neuroepithelial and pigment epithelial layers, which is more consistent with the manifestation of AMN. Additionally, the patients both had a significant central fixed black shadow and a significant decrease in visual acuity. Sheth et al^[16]^ administered systemic glucocorticoids and intravitreal injections of anti-angiogenic factors (ranibizumab) to patients with macular damage and achieved significant results. The 2 patients in this article improved symptomatically after oral administration of prednisolone tablets 30 mg, which confirms Sheth’s viewpoint.

COVID-19-induced macular damage involves a complex interplay of inflammatory, vascular, and neural factors. The elevated levels of inflammatory cytokines, such as interleukin-6 (IL-6) and tumor necrosis factor-alpha (TNF-α), contribute to retinal inflammation, disrupt the blood-retinal barrier (BRB), and impair the function of retinal pigment epithelial (RPE) cells. Inflammatory infiltration into the retinal tissue, alongside endothelial dysfunction, leads to vascular leakage, macular edema, and ischemia in the macular region. Additionally, the hypercoagulable state associated with COVID-19 may result in thrombosis in the retinal microvasculature, further compromising the retinal blood supply and contributing to neural damage.

In comparison to other retinal conditions, such as AMN and CRAO, several key distinctions can be made. AMN is primarily related to retinal microvascular dysfunction, with ischemia occurring in the deep retinal layers, often due to vasospasm or embolism, whereas the pathophysiology of COVID-19-induced macular damage is more multifactorial, involving significant inflammation, vascular dysfunction, and neural damage. CRAO, on the other hand, is typically characterized by acute arterial occlusion, leading to ischemia in the inner retinal layers and the classic “cherry-red” spot in the macular area. Unlike CRAO, which is a predominantly vascular occlusive event, COVID-19-induced macular damage presents with a broader range of inflammatory and vascular signs. These distinctions highlight the varied mechanisms by which COVID-19 can affect the retina, further emphasizing the need for tailored treatment approaches.

In the study of 2 cases of macular damage caused by COVID-19, it was found that both patients presented macular-related symptoms 3 days after the onset of COVID-19 symptoms. Fundus photography and macular OCT examinations revealed specific characteristics of macular lesions. After 1 week of systemic administration of low-to-medium doses of corticosteroids, the symptoms related to macular damage in the patients were alleviated. The visual acuity of 1 patient was significantly restored with a remarkable improvement in visual acuity test indicators, and the visual acuity of the other patient also improved to a certain extent. The macular structure showed a tendency for stabilization and improvement in the reexamined OCT images. This indicates that low-to-medium dose hormone therapy has a certain positive effect on macular damage caused by COVID-19 in the short term, which can prevent the disease from deteriorating and promote recovery to a certain extent. However, further research is still needed to determine the long-term effects and more precise treatment strategies.

In terms of long-term visual sequelae, it is important to consider that COVID-19-induced macular damage may lead to persistent visual impairment, including macular thinning, ongoing retinal inflammation, and potential atrophy. Given the multifaceted pathophysiology, the prognosis for visual recovery may be uncertain, and patients may require long-term monitoring to assess the risk of chronic damage and vision loss.

The study has several limitations. The sample size of merely 2 cases is very small, making it difficult to accurately represent the full spectrum of COVID-19-related macular diseases. The retrospective nature of the research may result in incomplete or inaccurate data from the medical records. The relatively short observation period means long-term visual function recovery and potential late complications remain unmonitored. Only 1 treatment approach, the systemic use of low-to-medium doses of corticosteroids, was employed, without comparison to other possible treatments. Additionally, individual differences like age and preexisting health conditions among patients could not be fully accounted for, which might have affected the precision of the conclusions about the disease and treatment outcomes.

## 4. Conclusion

Overall, the changes in the condition of the 2 patients in this report demonstrate that COVID-19 infection in the early stages (within 1 week) can directly cause macular disease, and this case report provides valuable information for treating COVID-19 retinal disease. This study carefully observes the changes in macular lesions in patients infected with novel coronavirus, analyzes and discusses the mechanisms and causes of macular damage, and explores possible treatment strategies and treatment timing. It assists in the early detection, early prevention, and systematic treatment of these patients, and has significance in improving the prognosis of novel coronavirus-related macular diseases.

## Author contributions

**Conceptualization:** Yan Li.

**Data curation:** Yong Guo.

**Investigation:** Yan Li, Xiaozhan Liu.

**Methodology:** Chenjun Guo, Yongkang Zhang.

**Resources:** Yong Guo, Yongkang Zhang.

**Software:** Chenjun Guo, Xiaozhan Liu.

**Supervision:** Houcheng Liang.

**Validation:** Houcheng Liang.

**Writing – original draft:** Yan Li, Yongkang Zhang.
